# Supplementary material for: Trends and biases in African large carnivore population assessments: identifying priorities and opportunities from a systematic review of two decades of research
Source: PeerJ. 2022 Nov 25;10:e14354. doi: 10.7717/peerj.14354 (PMC9703985; doi:10.7717/peerj.14354)
Supplement: Supplemental Information 2 [file peerj-10-14354-s002.docx]

**Systematic Review/Meta-Analysis Search**

Please clarify who are the two authors who performed the Search Strategy and identify them in your manuscript.

*Added information in line 113.*

You should also describe how disagreements were resolved, and identify the referee in your manuscript.

*There no disagreements in the process – this has now been stated in line 120. We have also added details on who carried out the screening process (*

**Systematic Review and/or Meta-Analysis Rationale**

For systematic reviews / meta-analyses, authors need to provide the following information:

The rationale for conducting the systematic review / meta-analysis;

*Have reworded slightly to explain the rationale behind this (line 95). Please also note that the PRISMA checklist is provided in Appendix S1.*

The contribution that it makes to knowledge in light of previously published related reports, including other meta-analyses and systematic reviews

*How the information was used is provided in line 101, and the whole Discussion section is the contribution to knowledge of this systematic review and meta-analyses. There had been no similar prior meta-analyses or systematic reviews.*
