# Supplementary material for: Trends and biases in African large carnivore population assessments: identifying priorities and opportunities from a systematic review of two decades of research
Source: PeerJ. 2022 Nov 25;10:e14354. doi: 10.7717/peerj.14354 (PMC9703985; doi:10.7717/peerj.14354)
Supplement: Supplemental Information 3 [file peerj-10-14354-s003.docx]

SUPPLEMENTARY INFORMATION

Appendix S1. PRISMA Checklist

Appendix S2. African Large Carnivore Density Studies & Estimates

Appendix S3. Additional Reporting Details and Methodological Considerations & Study Limitations

Appendix S4. Supplementary Tables & Figures

Appendix S5. Additional Model Fitting Details

Appendix S1 – PRISMA Checklist

| **Section and Topic** | **Item #** | **Checklist item** | **Page where item is reported** |
| --- | --- | --- | --- |
| **TITLE** | | |  |
| Title | 1 | Identify the report as a systematic review. | 1 |
| **ABSTRACT** | | |  |
| Abstract | 2 | See the PRISMA 2020 for Abstracts checklist. | 2 |
| **INTRODUCTION** | | |  |
| Rationale | 3 | Describe the rationale for the review in the context of existing knowledge. | 4 |
| Objectives | 4 | Provide an explicit statement of the objective(s) or question(s) the review addresses. | 5 |
| **METHODS** | | |  |
| Eligibility criteria | 5 | Specify the inclusion and exclusion criteria for the review and how studies were grouped for the syntheses. | 5 |
| Information sources | 6 | Specify all databases, registers, websites, organisations, reference lists and other sources searched or consulted to identify studies. Specify the date when each source was last searched or consulted. | 5 |
| Search strategy | 7 | Present the full search strategies for all databases, registers and websites, including any filters and limits used. | 5 |
| Selection process | 8 | Specify the methods used to decide whether a study met the inclusion criteria of the review, including how many reviewers screened each record and each report retrieved, whether they worked independently, and if applicable, details of automation tools used in the process. | 6 |
| Data collection process | 9 | Specify the methods used to collect data from reports, including how many reviewers collected data from each report, whether they worked independently, any processes for obtaining or confirming data from study investigators, and if applicable, details of automation tools used in the process. | 6 |
| Data items | 10a | List and define all outcomes for which data were sought. Specify whether all results that were compatible with each outcome domain in each study were sought (e.g. for all measures, time points, analyses), and if not, the methods used to decide which results to collect. | 6 |
|  | 10b | List and define all other variables for which data were sought (e.g. participant and intervention characteristics, funding sources). Describe any assumptions made about any missing or unclear information. | 6 |
| Study risk of bias assessment | 11 | Specify the methods used to assess risk of bias in the included studies, including details of the tool(s) used, how many reviewers assessed each study and whether they worked independently, and if applicable, details of automation tools used in the process. | 6 |
| Effect measures | 12 | Specify for each outcome the effect measure(s) (e.g. risk ratio, mean difference) used in the synthesis or presentation of results. | NA |
| Synthesis methods | 13a | Describe the processes used to decide which studies were eligible for each synthesis (e.g. tabulating the study intervention characteristics and comparing against the planned groups for each synthesis (item #5)). | 6 |
|  | 13b | Describe any methods required to prepare the data for presentation or synthesis, such as handling of missing summary statistics, or data conversions. | 7-9 |
|  | 13c | Describe any methods used to tabulate or visually display results of individual studies and syntheses. | 7-9 |
|  | 13d | Describe any methods used to synthesize results and provide a rationale for the choice(s). If meta-analysis was performed, describe the model(s), method(s) to identify the presence and extent of statistical heterogeneity, and software package(s) used. | 7-9 |
|  | 13e | Describe any methods used to explore possible causes of heterogeneity among study results (e.g. subgroup analysis, meta-regression). | 7-9 |
|  | 13f | Describe any sensitivity analyses conducted to assess robustness of the synthesized results. | NA |
| Reporting bias assessment | 14 | Describe any methods used to assess risk of bias due to missing results in a synthesis (arising from reporting biases). | 8-9 |
| Certainty assessment | 15 | Describe any methods used to assess certainty (or confidence) in the body of evidence for an outcome. | 8-9 |
| **RESULTS** | | |  |
| Study selection | 16a | Describe the results of the search and selection process, from the number of records identified in the search to the number of studies included in the review, ideally using a flow diagram. | Fig. 1 |
|  | 16b | Cite studies that might appear to meet the inclusion criteria, but which were excluded, and explain why they were excluded. | Fig. 1 |
| Study characteristics | 17 | Cite each included study and present its characteristics. | Appendix S2 |
| Risk of bias in studies | 18 | Present assessments of risk of bias for each included study. | NA |
| Results of individual studies | 19 | For all outcomes, present, for each study: (a) summary statistics for each group (where appropriate) and (b) an effect estimate and its precision (e.g. confidence/credible interval), ideally using structured tables or plots. | Figs. 3-5 |
| Results of syntheses | 20a | For each synthesis, briefly summarise the characteristics and risk of bias among contributing studies. | 10-12 |
|  | 20b | Present results of all statistical syntheses conducted. If meta-analysis was done, present for each the summary estimate and its precision (e.g. confidence/credible interval) and measures of statistical heterogeneity. If comparing groups, describe the direction of the effect. | 10-12 |
|  | 20c | Present results of all investigations of possible causes of heterogeneity among study results. | 10-12, Fig. 4 |
|  | 20d | Present results of all sensitivity analyses conducted to assess the robustness of the synthesized results. | NA |
| Reporting biases | 21 | Present assessments of risk of bias due to missing results (arising from reporting biases) for each synthesis assessed. | NA |
| Certainty of evidence | 22 | Present assessments of certainty (or confidence) in the body of evidence for each outcome assessed. | Fig. 4 |
| **DISCUSSION** | | |  |
| Discussion | 23a | Provide a general interpretation of the results in the context of other evidence. | 14-18 |
|  | 23b | Discuss any limitations of the evidence included in the review. | Appendix S3 |
|  | 23c | Discuss any limitations of the review processes used. | Appendix S3 |
|  | 23d | Discuss implications of the results for practice, policy, and future research. | Appendix S3 |
| **OTHER INFORMATION** | | |  |
| Registration and protocol | 24a | Provide registration information for the review, including register name and registration number, or state that the review was not registered. | NA |
|  | 24b | Indicate where the review protocol can be accessed, or state that a protocol was not prepared. | NA |
|  | 24c | Describe and explain any amendments to information provided at registration or in the protocol. | NA |
| Support | 25 | Describe sources of financial or non-financial support for the review, and the role of the funders or sponsors in the review. | Funders’ list |
| Competing interests | 26 | Declare any competing interests of review authors. | Competing interests statement |
| Availability of data, code and other materials | 27 | Report which of the following are publicly available and where they can be found: template data collection forms; data extracted from included studies; data used for all analyses; analytic code; any other materials used in the review. | Appendix S2 |

Appendix S2 – African large carnivore density studies & estimates

A list of all peer-reviewed density estimation studies from the literature review, as well as individual density estimates and additional information extracted during the review process, can be accessed at:

<https://github.com/pstrampelli/African-Large-Carnivore-Density-Studies-Estimates>

Appendix S3 – Additional Reporting Details and Methodological Considerations & Study Limitations

**Additional Reporting Details**

The details below are relevant to the data presented in Appendix S2.

In cases where a survey spanned multiple PAs and the overall combined size of the PAs sampled was unclear (e.g. Rich et al., 2019), the size of the largest of the PAs surveyed is reported.

When not explicitly presented, the area sampled by a study was calculated depending on the methodology employed: for studies employing camera trap grids, this was calculated as the area formed by the minimum convex polygon (MCP) between the different camera traps; for call-in studies, it consisted of the number of call-in stations multiplied by the area sampled by each (calculated as *π* multiplied by the square of the response radius (in km) employed by the authors); and for spoor counts, distance sampling, and intensive monitoring studies it consisted of the area provided by the authors. If the sampled area was not provided by the authors, or it was not possible to calculate it through the above methodology based on information provided by the authors, it was recorded as ‘Unclear’.

Where a single study provided two results for different years for the same exact study area (e.g. Rosenblatt et al., 2014) the most recent is provided. Similarly, for long-term intensive studies presenting a range of density estimates for the same area in a single study, the most recent is presented.

Where a minimum and a maximum density was provided for the study period, but no single average density or additional information was (e.g. M’soka et al., 2016), the estimate presented consists of the arithmetic mean of the minimum and maximum.

Note that, throughout this study, geographical regions and country boundaries are based on definitions by the African Union, as provided here: <https://web.archive.org/web/20130927110741/http://www.afrimap.org/english/images/report/AfriMAP-AU-Guide-EN.pdf>

**Methodological Considerations & Study Limitations**

Our review identified a number of density estimation efforts which were not published in peer-reviewed literature and/or were not publicly available. It is therefore important to acknowledge that the status of some populations has been assessed, and may be actively monitored, even though research findings are not available. In addition, in South Africa, several smaller protected areas hold populations of lion, cheetah and wild dog that are intensively monitored (Buk et al., 2018; Davies-Mostert et al., 2015), meaning that – although they do not qualify for this review – their status is known.

Thus, while our goal was to specifically identify biases and opportunities with regards to where exploratory peer-reviewed research is being carried out, it is clear that ours is an underestimate of the overall number of populations for which data on status and trends exists (particularly in southern Africa), and it is likely that consideration of grey literature would have influenced some of the conclusions of this study. Nonetheless, while we appreciate that subjecting estimates to peer-review may not be considered necessary if the sole goal is management of a population, for wider meta-analyses and policy, and to ensure their reliability and accordance with developed methodologies, population assessments benefit from undergoing scientific peer-review (Suryawanshi *et al.*, 2019). We therefore encourage researchers, managers, and practitioners to publish findings of population assessments whenever possible, to confirm their reliability and make insights available to the wider scientific research and conservation management communities.

In addition, although we did our best to correctly identify the nationality of the authors from an internet search, it is possible that accounting for certain factors (e.g. dual nationalities) might have led to slightly different results.

Appendix S4 – Supplementary Tables & Figures

| **Table S1.** Geographical trends in large African carnivore population assessments, by region (2000 – 2020). | | | | | | |
| --- | --- | --- | --- | --- | --- | --- |
| **Region** | **Geographical Range (km^2^)*** | | **Studies** | **Density Estimates** | **Studies / 10,000 km^2 **^** | **Estimates / 10,000 km^2 **^** |
| **LION (*Panthera leo*)** | | |  |  |  |  |
| Eastern | | 585,600 | 23 | 37 | 0.39 | 0.63 |
| Southern | | 737,450 | 20 | 34 | 0.27 | 0.46 |
| Western | | 52,300 | 4 | 5 | 0.76 | 0.96 |
| Central | | 299,140 | 6 | 14 | 0.20 | 0.47 |
| Northern | | 0 | 0 | 0 | NA | NA |
| *Total range* | | *1,654,375* |  |  |  |  |
| **LEOPARD (*Panthera pardus*)** | | |  |  |  |  |
| Southern | | 2,908,700 | 23 | 44 | 0.079 | 0.151 |
| Eastern | | 1,754,700 | 6 | 14 | 0.034 | 0.080 |
| Central | | 1,901,600 | 3 | 10 | 0.016 | 0.053 |
| Western | | 317,100 | 1 | 3 | 0.032 | 0.095 |
| Northern | | 9,400 | 0 | 0 | 0 | 0 |
| *Total range* | | *6,613,000* |  |  |  |  |
| **CHEETAH (*Acinonyx jubatus)*** | | |  |  |  |  |
| Southern | | 1,233,400 | 11 | 12 | 0.089 | 0.097 |
| Eastern | | 484,732 | 7 | 8 | 0.144 | 0.165 |
| Northern | | 704,300 | 1 | 1 | 0.014 | 0.014 |
| Western | | 99,440 | 0 | 0 | 0.000 | 0.000 |
| Central | | 238,300 | 0 | 0 | 0 | 0 |
| *Total range* | | *2,976,963* |  |  |  |  |
| **AFRICAN WILD DOG (*Lycaon pictus*)** | | | |  |  |  |
| Southern | | 693,400 | 3 | 3 | 0.043 | 0.043 |
| Eastern | | 369,526 | 2 | 2 | 0.054 | 0.054 |
| Central | | 156,740 | 1 | 2 | 0.064 | 0.128 |
| Western | | 29,200 | 0 | 0 | 0 | 0 |
| Northern | | 0 | 0 | 0 | NA | NA |
| *Total range* | | *1,303,469* |  |  |  |  |
| **SPOTTED HYAENA (*Crocuta crocuta*)** | | | |  |  |  |
| Southern | | 4,247,200 | 16 | 45 | 0.038 | 0.106 |
| Eastern | | 4,988,600 | 12 | 22 | 0.024 | 0.044 |
| Central | | 2,200,500 | 6 | 14 | 0.027 | 0.064 |
| Western | | 2,697,100 | 0 | 0 | 0 | 0 |
| Northern | | 110,960 | 0 | 0 | 0 | 0 |
| *Total range* | | *14,498,078* |  |  |  |  |
| **STRIPED HYAENA (*Hyaena hyaena*)** | | | |  |  |  |
| Eastern | | 4,404,298 | 3 | 3 | 0.0068 | 0.0068 |
| Western | | 1,637,400 | 0 | 0 | 0 | 0 |
| Central | | 1,320,300 | 0 | 0 | 0 | 0 |
| Northern | | 7,320,500 | 0 | 0 | 0 | 0 |
| Southern | | 0 | 0 | 0 | NA | NA |
| *Total range* | | *15,244,137* |  |  |  |  |
| **BROWN HYAENA (*Hyaena brunnea*)** | | | |  |  |  |
| Southern | | 2,294,769 | 12 | 39 | 0.052 | 0.170 |
| Eastern | | 0 | 0 | 0 | NA | NA |
| Western | | 0 | 0 | 0 | NA | NA |
| Central | | 0 | 0 | 0 | NA | NA |
| Northern | | 0 | 0 | 0 | NA | NA |
| *Total range* | | *2,294,769* |  |  |  |  |
| * Based on IUCN Red List geographical range polygons (IUCN, 2020)  ** Density of studies and estimates per 10,000 km^2^ of geographical range | | | | | | |

| **Table S2.** Peer-reviewed lion population assessments in Africa (2000-2020), by country. | | | | | |
| --- | --- | --- | --- | --- | --- |
| **Country** | **Geographical Range (km^2^)*** | **Studies** | **Density Estimates** | **Studies / 10,000 km^2^ **** | **Estimates / 10,000 km^2^ **** |
| Tanzania | 377,300 | 10 | 19 | 0.27 | 0.50 |
| Kenya | 69,500 | 9 | 12 | 1.29 | 1.73 |
| South Africa | 40,600 | 6 | 14 | 1.48 | 3.45 |
| Botswana | 201,450 | 5 | 6 | 0.25 | 0.30 |
| Cameroon | 19,740 | 5 | 11 | 2.53 | 5.57 |
| Zambia | 113,800 | 4 | 8 | 0.35 | 0.70 |
| Zimbabwe | 44,400 | 3 | 3 | 0.68 | 0.68 |
| Uganda | 2,500 | 2 | 4 | 8.00 | 16.00 |
| Mozambique | 192,300 | 2 | 2 | 0.10 | 0.10 |
| Senegal | 18,300 | 2 | 2 | 1.09 | 1.09 |
| CAR | 264,100 | 1 | 3 | 0.04 | 0.11 |
| Nigeria | 7,400 | 1 | 2 | 1.35 | 2.70 |
| Benin | 13,000 | 1 | 1 | 0.77 | 0.77 |
| Burkina Faso | 10,600 | 1 | 1 | 0.94 | 0.94 |
| Ethiopia | 109,600 | 1 | 1 | 0.09 | 0.09 |
| Namibia | 128,700 | 1 | 1 | 0.08 | 0.08 |
| Sudan | 8,500 | 1 | 1 | 1.18 | 1.18 |
| Angola | 10,100 | 0 | 0 | 0.00 | 0.00 |
| Chad | 3,100 | 0 | 0 | 0.00 | 0.00 |
| DRC | 12,200 | 0 | 0 | 0.00 | 0.00 |
| Malawi | 6,100 | 0 | 0 | 0.00 | 0.00 |
| Niger | 3,000 | 0 | 0 | 0.00 | 0.00 |
| South Sudan | 18,200 | 0 | 0 | 0.00 | 0.00 |
| * Based on IUCN Red List geographical range polygons (IUCN, 2020)  ** Density of studies and estimates per 10,000 km^2^ of geographical range | | | | | |

| **Table S3.** Peer-reviewed leopard population assessments in Africa (2000-2020), by country. | | | | | |
| --- | --- | --- | --- | --- | --- |
| **Country** | **Geographical Range (km^2^)*** | **Studies** | **Density Estimates** | **Studies / 10,000 km^2^ **** | **Estimates / 10,000 km^2^ **** |
| South Africa | 401,300 | 14 | 28 | 0.35 | 0.70 |
| Tanzania | 672,100 | 4 | 12 | 0.06 | 0.18 |
| Zimbabwe | 160,000 | 2 | 3 | 0.13 | 0.19 |
| Cameroon | 184,300 | 2 | 8 | 0.11 | 0.43 |
| Kenya | 312,900 | 2 | 2 | 0.06 | 0.06 |
| Botswana | 367,200 | 2 | 4 | 0.05 | 0.11 |
| Namibia | 568,900 | 2 | 3 | 0.04 | 0.05 |
| Gabon | 250,000 | 1 | 3 | 0.04 | 0.12 |
| Malawi | 11,100 | 1 | 3 | 0.90 | 2.70 |
| Zambia | 236,100 | 1 | 2 | 0.04 | 0.08 |
| CAR | 375,500 | 1 | 2 | 0.03 | 0.05 |
| Mozambique | 457,000 | 1 | 1 | 0.02 | 0.02 |
| Angola | 696,900 | 0 | 0 | 0.00 | 0.00 |
| DRC | 696,600 | 0 | 0 | 0.00 | 0.00 |
| Ethiopia | 346,900 | 0 | 0 | 0.00 | 0.00 |
| Congo | 310,300 | 0 | 0 | 0.00 | 0.00 |
| South Sudan | 249,800 | 0 | 0 | 0.00 | 0.00 |
| Benin | 132,300 | 0 | 0 | 0.00 | 0.00 |
| Chad | 71,200 | 0 | 0 | 0.00 | 0.00 |
| Uganda | 65,100 | 0 | 0 | 0.00 | 0.00 |
| Sudan | 42,200 | 0 | 0 | 0.00 | 0.00 |
| Somalia | 39,300 | 0 | 0 | 0.00 | 0.00 |
| Cote d'Ivoire | 39,200 | 0 | 0 | 0.00 | 0.00 |
| Guinea | 31,200 | 0 | 0 | 0.00 | 0.00 |
| Senegal | 29,400 | 0 | 0 | 0.00 | 0.00 |
| Liberia | 23,000 | 0 | 0 | 0.00 | 0.00 |
| Eritrea | 22,600 | 0 | 0 | 0.00 | 0.00 |
| Burkina Faso | 19,000 | 0 | 0 | 0.00 | 0.00 |
| Ghana | 14,700 | 0 | 0 | 0.00 | 0.00 |
| Equatorial Guinea | 12,800 | 0 | 0 | 0.00 | 0.00 |
| Nigeria | 11,500 | 0 | 0 | 0.00 | 0.00 |
| Eswatini | 10,100 | 0 | 0 | 0.00 | 0.00 |
| Guinea-Bissau | 7,000 | 0 | 0 | 0.00 | 0.00 |
| Mali | 6,000 | 0 | 0 | 0.00 | 0.00 |
| Egypt | 5,800 | 0 | 0 | 0.00 | 0.00 |
| Algeria | 3,600 | 0 | 0 | 0.00 | 0.00 |
| Sierra Leone | 3,000 | 0 | 0 | 0.00 | 0.00 |
| Rwanda | 2,200 | 0 | 0 | 0.00 | 0.00 |
| Djibouti | 1,600 | 0 | 0 | 0.00 | 0.00 |
| Burundi | 900 | 0 | 0 | 0.00 | 0.00 |
| Niger | 500 | 0 | 0 | 0.00 | 0.00 |
| Togo | 300 | 0 | 0 | 0.00 | 0.00 |
| Lesotho | 100 | 0 | 0 | 0.00 | 0.00 |
| * Based on IUCN Red List geographical range polygons (IUCN, 2020)  ** Density of studies and estimates per 10,000 km^2^ of geographical range | | | | | |

| **Table S4.** Peer-reviewed cheetah population assessments in Africa (2000-2020), by country. | | | | | |
| --- | --- | --- | --- | --- | --- |
| **Country** | **Geographical Range (km^2^)*** | **Studies** | **Density Estimates** | **Studies / 10,000 km^2^ **** | **Estimates / 10,000 km^2^ **** |
| Botswana | 453,000 | 5 | 7 | 0.11 | 0.15 |
| Kenya | 123,800 | 5 | 5 | 0.40 | 0.40 |
| Namibia | 510,000 | 2 | 2 | 0.04 | 0.04 |
| Tanzania | 119,000 | 2 | 3 | 0.17 | 0.25 |
| Algeria | 704,300 | 1 | 1 | 0.01 | 0.01 |
| South Africa | 140,300 | 1 | 1 | 0.07 | 0.07 |
| Zimbabwe | 48,100 | 1 | 1 | 0.21 | 0.21 |
| Zambia | 29,400 | 1 | 1 | 0.34 | 0.34 |
| Chad | 204,200 | 0 | 0 | 0.00 | 0.00 |
| Ethiopia | 193,392 | 0 | 0 | 0.00 | 0.00 |
| Mali | 63,300 | 0 | 0 | 0.00 | 0.00 |
| South Sudan | 44,740 | 0 | 0 | 0.00 | 0.00 |
| Angola | 44,700 | 0 | 0 | 0.00 | 0.00 |
| CAR | 34,100 | 0 | 0 | 0.00 | 0.00 |
| Niger | 13,690 | 0 | 0 | 0.00 | 0.00 |
| Benin | 13,100 | 0 | 0 | 0.00 | 0.00 |
| Burkina Faso | 9,350 | 0 | 0 | 0.00 | 0.00 |
| Mozambique | 7,900 | 0 | 0 | 0.00 | 0.00 |
| Uganda | 2,600 | 0 | 0 | 0.00 | 0.00 |
| Sudan | 1,200 | 0 | 0 | 0.00 | 0.00 |
| * Based on IUCN Red List geographical range polygons (IUCN, 2020)  ** Density of studies and estimates per 10,000 km^2^ of geographical range | | | | | |

| **Table S5.** Peer-reviewed African wild dog population assessments in Africa (2000-2020), by country. | | | | | |
| --- | --- | --- | --- | --- | --- |
| **Country** | **Geographical Range (km^2^)*** | **Studies** | **Density Estimates** | **Studies / 10,000 km^2^ **** | **Estimates / 10,000 km^2^ **** |
| Kenya | 68,300 | 2 | 2 | 0.29 | 0.29 |
| South Africa | 22,900 | 2 | 2 | 0.87 | 0.87 |
| CAR | 60,740 | 1 | 2 | 0.16 | 0.33 |
| Zimbabwe | 38,000 | 1 | 1 | 0.26 | 0.26 |
| Botswana | 352,400 | 0 | 0 | 0.00 | 0.00 |
| Tanzania | 213,300 | 0 | 0 | 0.00 | 0.00 |
| Zambia | 173,100 | 0 | 0 | 0.00 | 0.00 |
| Chad | 88,800 | 0 | 0 | 0.00 | 0.00 |
| Namibia | 84,100 | 0 | 0 | 0.00 | 0.00 |
| South Sudan | 41,732 | 0 | 0 | 0.00 | 0.00 |
| Ethiopia | 30,594 | 0 | 0 | 0.00 | 0.00 |
| Mozambique | 22,900 | 0 | 0 | 0.00 | 0.00 |
| Sudan | 15,600 | 0 | 0 | 0.00 | 0.00 |
| Nigeria | 11,600 | 0 | 0 | 0.00 | 0.00 |
| Senegal | 8,400 | 0 | 0 | 0.00 | 0.00 |
| Cameroon | 7,200 | 0 | 0 | 0.00 | 0.00 |
| Burkina Faso | 6,400 | 0 | 0 | 0.00 | 0.00 |
| Benin | 2,800 | 0 | 0 | 0.00 | 0.00 |
| * Based on IUCN Red List geographical range polygons (IUCN, 2020)  ** Density of studies and estimates per 10,000 km^2^ of geographical range | | | | | |

| **Table S6.** Peer-reviewed spotted hyaena population assessments in Africa (2000-2020), by country. | | | | | |
| --- | --- | --- | --- | --- | --- |
| **Country** | **Geographical Range (km^2^)*** | **Studies** | **Density Estimates** | **Studies / 10,000 km^2^ **** | **Estimates / 10,000 km^2^ **** |
| Cameroon | 172,100 | 4 | 10 | 0.23 | 0.58 |
| South Africa | 220,100 | 4 | 24 | 0.18 | 1.09 |
| Namibia | 368,500 | 4 | 9 | 0.11 | 0.24 |
| Kenya | 580,400 | 4 | 7 | 0.07 | 0.12 |
| Ethiopia | 1,104,000 | 4 | 4 | 0.04 | 0.04 |
| Botswana | 552,100 | 3 | 3 | 0.05 | 0.05 |
| Tanzania | 945,100 | 3 | 7 | 0.03 | 0.07 |
| Zimbabwe | 297,400 | 2 | 4 | 0.07 | 0.13 |
| Mozambique | 711,100 | 2 | 2 | 0.03 | 0.03 |
| Uganda | 241,000 | 1 | 3 | 0.04 | 0.12 |
| CAR | 405,900 | 1 | 3 | 0.02 | 0.07 |
| Malawi | 118,400 | 1 | 3 | 0.08 | 0.25 |
| Congo | 75,700 | 1 | 1 | 0.13 | 0.13 |
| Sudan | 716,800 | 1 | 1 | 0.01 | 0.01 |
| Zambia | 752,600 | 1 | 1 | 0.01 | 0.01 |
| Angola | 1,214,600 | 0 | 0 | 0.00 | 0.00 |
| DRC | 1,042,200 | 0 | 0 | 0.00 | 0.00 |
| Nigeria | 770,500 | 0 | 0 | 0.00 | 0.00 |
| Somalia | 637,700 | 0 | 0 | 0.00 | 0.00 |
| South Sudan | 619,700 | 0 | 0 | 0.00 | 0.00 |
| Mali | 513,700 | 0 | 0 | 0.00 | 0.00 |
| Chad | 476,800 | 0 | 0 | 0.00 | 0.00 |
| Burkina Faso | 274,200 | 0 | 0 | 0.00 | 0.00 |
| Guinea | 245,900 | 0 | 0 | 0.00 | 0.00 |
| Cote d'Ivoire | 231,200 | 0 | 0 | 0.00 | 0.00 |
| Senegal | 196,700 | 0 | 0 | 0.00 | 0.00 |
| Ghana | 166,800 | 0 | 0 | 0.00 | 0.00 |
| Eritrea | 117,600 | 0 | 0 | 0.00 | 0.00 |
| Benin | 115,100 | 0 | 0 | 0.00 | 0.00 |
| Mauritania | 110,960 | 0 | 0 | 0.00 | 0.00 |
| Sierra Leone | 59,700 | 0 | 0 | 0.00 | 0.00 |
| Togo | 52,200 | 0 | 0 | 0.00 | 0.00 |
| Guinea-Bissau | 36,000 | 0 | 0 | 0.00 | 0.00 |
| Burundi | 27,800 | 0 | 0 | 0.00 | 0.00 |
| Rwanda | 26,300 | 0 | 0 | 0.00 | 0.00 |
| Liberia | 18,200 | 0 | 0 | 0.00 | 0.00 |
| Eswatini | 12,400 | 0 | 0 | 0.00 | 0.00 |
| The Gambia | 11,300 | 0 | 0 | 0.00 | 0.00 |
| Gabon | 5,600 | 0 | 0 | 0.00 | 0.00 |
| * Based on IUCN Red List geographical range polygons (IUCN, 2020)  ** Density of studies and estimates per 10,000 km^2^ of geographical range | | | | | |

| **Table S7.** Peer-reviewed striped hyaena population assessments in Africa (2000-2020), by country. | | | | | |
| --- | --- | --- | --- | --- | --- |
| **Country** | **Geographical Range (km^2^)*** | **Studies** | **Density Estimates** | **Studies / 10,000 km^2^ **** | **Estimates / 10,000 km^2^ **** |
| Kenya | 580,400 | 3 | 3 | 0.05 | 0.05 |
| Cameroon | 217,400 | 0 | 0 | 0 | 0 |
| Chad | 1,096,200 | 0 | 0 | 0 | 0 |
| CAR | 6,700 | 0 | 0 | 0 | 0 |
| Eritrea | 117,598 | 0 | 0 | 0 | 0 |
| Djibouti | 23,200 | 0 | 0 | 0 | 0 |
| Somalia | 637,700 | 0 | 0 | 0 | 0 |
| Sudan | 945,100 | 0 | 0 | 0 | 0 |
| South Sudan | 297,700 | 0 | 0 | 0 | 0 |
| Uganda | 157,700 | 0 | 0 | 0 | 0 |
| Tanzania | 544,900 | 0 | 0 | 0 | 0 |
| Ethiopia | 1,100,000 | 0 | 0 | 0 | 0 |
| Morocco | 976,900 | 0 | 0 | 0 | 0 |
| Algeria | 2,382,000 | 0 | 0 | 0 | 0 |
| Tunisia | 163,600 | 0 | 0 | 0 | 0 |
| Libya | 1,758,000 | 0 | 0 | 0 | 0 |
| Egypt | 1,010,000 | 0 | 0 | 0 | 0 |
| Mauritania | 1,030,000 | 0 | 0 | 0 | 0 |
| Senegal | 97,000 | 0 | 0 | 0 | 0 |
| Mali | 1,019,600 | 0 | 0 | 0 | 0 |
| Burkina Faso | 253,000 | 0 | 0 | 0 | 0 |
| Ghana | 27,600 | 0 | 0 | 0 | 0 |
| Togo | 2,100 | 0 | 0 | 0 | 0 |
| Benin | 24,100 | 0 | 0 | 0 | 0 |
| Nigeria | 211,000 | 0 | 0 | 0 | 0 |
| Cote d'Ivoire | 3,000 | 0 | 0 | 0 | 0 |
| * Based on IUCN Red List geographical range polygons (IUCN, 2020)  ** Density of studies and estimates per 10,000 km^2^ of geographical range | | | | | |

| **Table S8.** Peer-reviewed brown hyaena population assessments in Africa (2000-2020), by country. | | | | | |
| --- | --- | --- | --- | --- | --- |
| **Country** | **Geographical Range (km^2^)*** | **Studies** | **Density Estimates** | **Studies / 10,000 km^2^ **** | **Estimates / 10,000 km^2^ **** |
| South Africa | 776,700 | 5 | 19 | 0.06 | 0.24 |
| Botswana | 543,000 | 4 | 18 | 0.07 | 0.33 |
| Namibia | 728,400 | 1 | 1 | 0.01 | 0.01 |
| Zimbabwe | 164,900 | 1 | 1 | 0.06 | 0.06 |
| Angola | 42,900 | 0 | 0 | 0.00 | 0.00 |
| Mozambique | 22,100 | 0 | 0 | 0.00 | 0.00 |
| Eswatini | 15,869 | 0 | 0 | 0.00 | 0.00 |
| Zambia | 900 | 0 | 0 | 0.00 | 0.00 |
| * Based on IUCN Red List geographical range polygons (IUCN, 2020)  ** Density of studies and estimates per 10,000 km^2^ of geographical range | | | | | |

| **Table S9.** Results of the GLMM investigating biases in large carnivore population assessments in Africa, by species, not adjusting for geographical range (Model 1). Species and countries are modelled as random effects. For both species and countries, a positive value indicates more assessments than expected, and a negative fewer. Values credibly different from zero (i.e. 95% highest density interval (HDI) of the posterior distribution does not contain zero) are highlighted in bold. | | | | |
| --- | --- | --- | --- | --- |
| **Name** | **Mean** | **HDI low** | **HDI high** | **SD** |
| Intercept | -2.13 | -3.48 | -0.87 | 0.67 |
| Country Random Effect Variance | 1.88 | 1.20 | 2.71 | 0.40 |
| Species Random Effect Variance | 1.16 | 0.41 | 2.21 | 0.56 |
| Brown Hyaena | 0.16 | -0.96 | 1.27 | 0.56 |
| Cheetah | 0.07 | -0.97 | 1.14 | 0.54 |
| Leopard | 0.44 | -0.57 | 1.50 | 0.52 |
| **Lion *** | **1.07** | **0.08** | **2.12** | **0.52** |
| Spotted Hyaena | 0.56 | -0.46 | 1.61 | 0.52 |
| **Striped Hyaena ^†^** | **-1.32** | **-2.78** | **-0.06** | **0.70** |
| African Wild Dog | -0.96 | -2.15 | 0.18 | 0.59 |
| South Africa * | 3.35 | 2.43 | 4.33 | 0.49 |
| Kenya * | 3.16 | 2.21 | 4.17 | 0.50 |
| Tanzania * | 2.91 | 1.94 | 3.95 | 0.52 |
| Botswana * | 2.81 | 1.82 | 3.83 | 0.51 |
| Cameroon * | 2.47 | 1.40 | 3.58 | 0.55 |
| Namibia * | 2.12 | 1.03 | 3.24 | 0.56 |
| Zimbabwe * | 2.12 | 1.01 | 3.22 | 0.56 |
| Zambia * | 1.73 | 0.53 | 2.90 | 0.60 |
| Ethiopia * | 1.46 | 0.19 | 2.74 | 0.65 |
| Mozambique * | 1.36 | 0.10 | 2.64 | 0.65 |
| CAR | 1.21 | -0.17 | 2.56 | 0.69 |
| Uganda | 0.93 | -0.56 | 2.40 | 0.75 |
| Malawi | 0.66 | -1.06 | 2.27 | 0.85 |
| Senegal | 0.56 | -1.11 | 2.19 | 0.84 |
| Nigeria | 0.49 | -1.64 | 2.57 | 1.08 |
| Algeria | 0.47 | -1.72 | 2.48 | 1.08 |
| Sudan | 0.44 | -1.22 | 2.06 | 0.83 |
| Congo | 0.40 | -1.77 | 2.37 | 1.06 |
| Gabon | 0.40 | -1.74 | 2.39 | 1.06 |
| Benin **^†^** | -0.24 | -2.29 | 1.60 | 0.99 |
| Burkina Faso **^†^** | -0.24 | -2.25 | 1.61 | 0.99 |
| Tunisia **^†^** | -0.31 | -3.67 | 3.10 | 1.73 |
| Libya **^†^** | -0.32 | -3.78 | 2.98 | 1.73 |
| Morocco **^†^** | -0.32 | -3.74 | 2.96 | 1.72 |
| Equatorial Guinea **^†^** | -0.77 | -3.82 | 2.13 | 1.55 |
| Lesotho **^†^** | -0.77 | -3.91 | 2.08 | 1.55 |
| The Gambia **^†^** | -0.81 | -4.00 | 1.92 | 1.54 |
| Egypt **^†^** | -0.83 | -3.88 | 2.00 | 1.53 |
| Djibouti **^†^** | -0.84 | -3.91 | 2.00 | 1.53 |
| Mauritania **^†^** | -0.87 | -3.89 | 1.95 | 1.52 |
| Guinea **^†^** | -1.07 | -3.99 | 1.59 | 1.46 |
| Guinea-Bissau **^†^** | -1.07 | -4.02 | 1.55 | 1.46 |
| Liberia **^†^** | -1.07 | -4.07 | 1.56 | 1.47 |
| Sierra Leone **^†^** | -1.07 | -3.99 | 1.64 | 1.46 |
| Burundi **^†^** | -1.08 | -4.05 | 1.60 | 1.46 |
| Rwanda **^†^** | -1.08 | -4.03 | 1.60 | 1.47 |
| Cote d'Ivoire **^†^** | -1.10 | -3.99 | 1.59 | 1.45 |
| Eritrea **^†^** | -1.11 | -4.01 | 1.57 | 1.46 |
| Ghana **^†^** | -1.11 | -4.00 | 1.56 | 1.44 |
| Somalia **^†^** | -1.11 | -4.06 | 1.52 | 1.46 |
| Togo **^†^** | -1.11 | -4.04 | 1.57 | 1.45 |
| Nigeria **^†^** | -1.15 | -3.99 | 1.54 | 1.44 |
| Eswatini **^†^** | -1.21 | -4.14 | 1.36 | 1.43 |
| Mali **^†^** | -1.23 | -4.11 | 1.33 | 1.42 |
| Niger **^†^** | -1.31 | -4.05 | 1.30 | 1.39 |
| DRC **^†^** | -1.36 | -4.13 | 1.20 | 1.39 |
| Chad **^†^** | -1.49 | -4.22 | 0.96 | 1.36 |
| South Sudan **^†^** | -1.49 | -4.24 | 0.98 | 1.36 |
| Angola **^†^** | -1.53 | -4.24 | 0.95 | 1.35 |
| **Legend:** HDI = Highest Density Interval; SD = standard deviation; * = value suggests significant positive effect; ^†^ = value suggests significant negative effect. | | | | |

| **Table S10.** Results of the models investigating biases in leopard population assessments in Africa, accounting for geographical range (Poisson GLM). One outlier (South Africa) was removed (strong positive effect). Significant (i.e. credible non-zero) fixed effect values are highlighted in bold. For residuals of individual countries, a positive value indicates more assessments than expected after controlling for the relevant variables, a negative value fewer. Values credibly different from zero (i.e. 95% highest density interval (HDI) of the posterior distribution does not contain zero) are highlighted in bold. | | | |
| --- | --- | --- | --- |
| **Name** | **Mean** | **HDI low** | **HDI high** |
| (Intercept) | -1.28 | -1.94 | -0.68 |
| **Range *** | **0.76** | **0.38** | **1.15** |
| GDP | NA | NA | NA |
| Frag | NA | NA | NA |
| **Zimbabwe *** | **3.32** | **2.05** | **4.84** |
| **Cameroon *** | **3.13** | **1.93** | **4.51** |
| **Kenya *** | **2.23** | **1.36** | **3.20** |
| **Botswana *** | **1.89** | **1.07** | **2.75** |
| **Zambia *** | **1.07** | **0.45** | **1.73** |
| Tanzania | 1.02 | -0.27 | 2.33 |
| **Gabon *** | **1.01** | **0.40** | **1.65** |
| Namibia | 0.77 | -0.07 | 1.67 |
| **CAR *** | **0.53** | **0.04** | **1.06** |
| Mozambique | 0.23 | -0.28 | 0.75 |
| **Lesotho ^†^** | **-0.41** | **-0.58** | **-0.24** |
| **Togo ^†^** | **-0.41** | **-0.58** | **-0.24** |
| **Niger ^†^** | **-0.41** | **-0.58** | **-0.24** |
| **Burundi ^†^** | **-0.41** | **-0.58** | **-0.24** |
| **Djibouti ^†^** | **-0.41** | **-0.58** | **-0.24** |
| **Rwanda ^†^** | **-0.41** | **-0.58** | **-0.24** |
| **Sierra Leone ^†^** | **-0.41** | **-0.58** | **-0.24** |
| **Algeria ^†^** | **-0.41** | **-0.58** | **-0.25** |
| **Egypt ^†^** | **-0.41** | **-0.58** | **-0.25** |
| **Mali ^†^** | **-0.41** | **-0.58** | **-0.25** |
| **Guinea-Bissau ^†^** | **-0.41** | **-0.58** | **-0.25** |
| **Eswatini ^†^** | **-0.41** | **-0.58** | **-0.25** |
| **Malawi ^†^** | **-0.41** | **-0.58** | **-0.25** |
| **Nigeria ^†^** | **-0.41** | **-0.58** | **-0.25** |
| **Equatorial Guinea ^†^** | **-0.41** | **-0.59** | **-0.25** |
| **Ghana ^†^** | **-0.42** | **-0.59** | **-0.25** |
| **Burkina Faso ^†^** | **-0.42** | **-0.59** | **-0.26** |
| **Liberia ^†^** | **-0.42** | **-0.59** | **-0.26** |
| **Senegal ^†^** | **-0.43** | **-0.60** | **-0.26** |
| **Guinea ^†^** | **-0.43** | **-0.60** | **-0.26** |
| **Cote d'Ivoire ^†^** | **-0.43** | **-0.60** | **-0.27** |
| **Sudan ^†^** | **-0.44** | **-0.61** | **-0.27** |
| **Uganda ^†^** | **-0.45** | **-0.62** | **-0.29** |
| **Chad ^†^** | **-0.46** | **-0.63** | **-0.29** |
| **Benin ^†^** | **-0.51** | **-0.68** | **-0.35** |
| **South Sudan ^†^** | **-0.62** | **-0.79** | **-0.45** |
| **Congo ^†^** | **-0.69** | **-0.87** | **-0.52** |
| **Ethiopia ^†^** | **-0.74** | **-0.92** | **-0.56** |
| **DRC ^†^** | **-1.39** | **-1.89** | **-0.89** |
| **Angola ^†^** | **-1.39** | **-1.89** | **-0.89** |
| **Legend:** HDI = Highest Density Interval; Range = Geographical range of species, based on IUCN Red List range maps; * = value suggests significant positive effect; ^†^ = value suggests significant negative effect. | | | |

| **Table S11.** Results of the models investigating biases in cheetah population assessments in Africa, accounting for geographical range (Poisson GLM). Significant (i.e. credible non-zero) fixed effect values are highlighted in bold. For residuals of individual countries, a positive value indicates more assessments than expected after controlling for the relevant variables, a negative value fewer. Values credibly different from zero (i.e. 95% highest density interval (HDI) of the posterior distribution does not contain zero) are highlighted in bold. | | | | | |
| --- | --- | --- | --- | --- | --- |
| **Name** | **Mean** | **HDI low** | **HDI high** | **Mean** | **HDI low** |
| (Intercept) | -0.49 | -1.09 | 0.09 | -1.51 | -2.65 |
| **Range *** | **0.56** | **0.19** | **0.92** | 0.10 | -0.58 |
| GDP | NA | NA | NA | 0.10 | -0.89 |
| Frag | NA | NA | NA | -1.24 | -2.79 |
| **Kenya *** | **3.18** | **1.80** | **4.63** | NA | NA |
| **Botswana *** | **2.86** | **1.35** | **4.44** | 0.39 | -1.52 |
| **Tanzania *** | **1.89** | **0.87** | **2.94** | **2.33** | **0.24** |
| **Zambia *** | **0.84** | **0.06** | **1.63** | **1.45** | **0.18** |
| **Zimbabwe *** | **0.78** | **0.06** | **1.56** | **3.60** | **0.50** |
| South Africa | 0.49 | -0.09 | 1.13 | -0.14 | -1.23 |
| Namibia | 0.16 | -0.76 | 1.08 | 0.02 | -1.19 |
| **Sudan ^†^** | **-0.65** | **-0.90** | **-0.42** | **-0.25** | **-0.49** |
| **Uganda ^†^** | **-0.66** | **-0.90** | **-0.42** | **-0.39** | **-0.62** |
| **Mozambique ^†^** | **-0.66** | **-0.90** | **-0.42** | **-0.40** | **-0.64** |
| **Burkina Faso ^†^** | **-0.66** | **-0.90** | **-0.42** | **-0.51** | **-0.79** |
| **Benin ^†^** | **-0.67** | **-0.91** | **-0.43** | **-0.92** | **-1.65** |
| **Niger ^†^** | **-0.67** | **-0.90** | **-0.43** | **-0.35** | **-0.58** |
| **CAR ^†^** | **-0.68** | **-0.92** | **-0.45** | **-0.23** | **-0.48** |
| **Angola ^†^** | **-0.69** | **-0.93** | **-0.46** | **-0.51** | **-0.83** |
| **South Sudan ^†^** | **-0.69** | **-0.93** | **-0.46** | **-0.22** | **-0.51** |
| **Mali ^†^** | **-0.71** | **-0.95** | **-0.48** | **-0.35** | **-0.59** |
| **Ethiopia ^†^** | **-0.86** | **-1.09** | **-0.63** | **-0.38** | **-0.63** |
| **Chad ^†^** | **-0.87** | **-1.10** | **-0.64** | **-0.26** | **-0.53** |
| **Algeria ^†^** | **-1.23** | **-2.30** | **-0.15** | 0.06 | -1.57 |
| **Legend:** HDI = Highest Density Interval; Range = Geographical range of species, based on IUCN Red List range maps; * = value suggests significant positive effect; ^†^ = value suggests significant negative effect. | | | | | |

| **Table S12.** Results of the models investigating biases in spotted hyaena population assessments in Africa, accounting for geographical range (Poisson GLM). Significant (i.e. credible non-zero) fixed effect values are highlighted in bold. For residuals of individual countries, a positive value indicates more assessments than expected after controlling for the relevant variables, a negative value fewer. Values credibly different from zero (i.e. 95% highest density interval (HDI) of the posterior distribution does not contain zero) are highlighted in bold. | | | |
| --- | --- | --- | --- |
| **Name** | **Mean** | **HDI low** | **HDI high** |
| (Intercept) | -0.18 | -0.56 | 0.17 |
| **Range *** | **0.41** | **0.11** | **0.71** |
| GDP | NA | NA | NA |
| Frag | NA | NA | NA |
| **Cameroon *** | **4.13** | **2.85** | **5.48** |
| **South Africa *** | **3.96** | **2.83** | **5.26** |
| **Namibia *** | **3.47** | **2.50** | **4.44** |
| **Kenya *** | **2.81** | **2.02** | **3.69** |
| **Botswana *** | **1.92** | **1.28** | **2.62** |
| **Zimbabwe *** | **1.41** | **0.84** | **2.08** |
| **Ethiopia *** | **1.40** | **0.13** | **2.71** |
| **Tanzania *** | **1.03** | **0.15** | **1.95** |
| **Mozambique *** | **0.65** | **0.11** | **1.21** |
| Congo | 0.54 | 0.00 | 1.08 |
| Uganda | 0.33 | -0.07 | 0.78 |
| CAR | 0.13 | -0.23 | 0.48 |
| Sudan | -0.24 | -0.61 | 0.15 |
| Zambia | -0.29 | -0.68 | 0.11 |
| **Gabon ^†^** | **-0.74** | **-0.95** | **-0.53** |
| **The Gambia ^†^** | **-0.75** | **-0.95** | **-0.54** |
| **Eswatini ^†^** | **-0.75** | **-0.95** | **-0.54** |
| **Liberia ^†^** | **-0.75** | **-0.96** | **-0.55** |
| **Rwanda ^†^** | **-0.75** | **-0.96** | **-0.55** |
| **Burundi ^†^** | **-0.75** | **-0.96** | **-0.55** |
| **Guinea-Bissau ^†^** | **-0.76** | **-0.96** | **-0.56** |
| **Togo ^†^** | **-0.76** | **-0.97** | **-0.56** |
| **Sierra Leone ^†^** | **-0.77** | **-0.97** | **-0.57** |
| **Mauritania ^†^** | **-0.79** | **-0.98** | **-0.60** |
| **Benin ^†^** | **-0.79** | **-0.99** | **-0.60** |
| **Malawi ^†^** | **-0.79** | **-0.99** | **-0.60** |
| **Ghana ^†^** | **-0.82** | **-1.00** | **-0.63** |
| **Senegal ^†^** | **-0.83** | **-1.01** | **-0.65** |
| **Cote d'Ivoire ^†^** | **-0.85** | **-1.02** | **-0.67** |
| **Guinea ^†^** | **-0.86** | **-1.03** | **-0.68** |
| **Burkina Faso ^†^** | **-0.87** | **-1.04** | **-0.70** |
| **Chad ^†^** | **-0.98** | **-1.14** | **-0.81** |
| **Mali ^†^** | **-1.00** | **-1.17** | **-0.84** |
| **South Sudan ^†^** | **-1.07** | **-1.26** | **-0.89** |
| **Nigeria ^†^** | **-1.17** | **-1.41** | **-0.94** |
| **DRC ^†^** | **-1.39** | **-1.78** | **-1.02** |
| **Angola ^†^** | **-1.55** | **-2.10** | **-1.06** |
| **Legend:** HDI = Highest Density Interval; Range = Geographical range of species, based on IUCN Red List range maps; * = value suggests significant positive effect; ^†^ = value suggests significant negative effect. | | | |


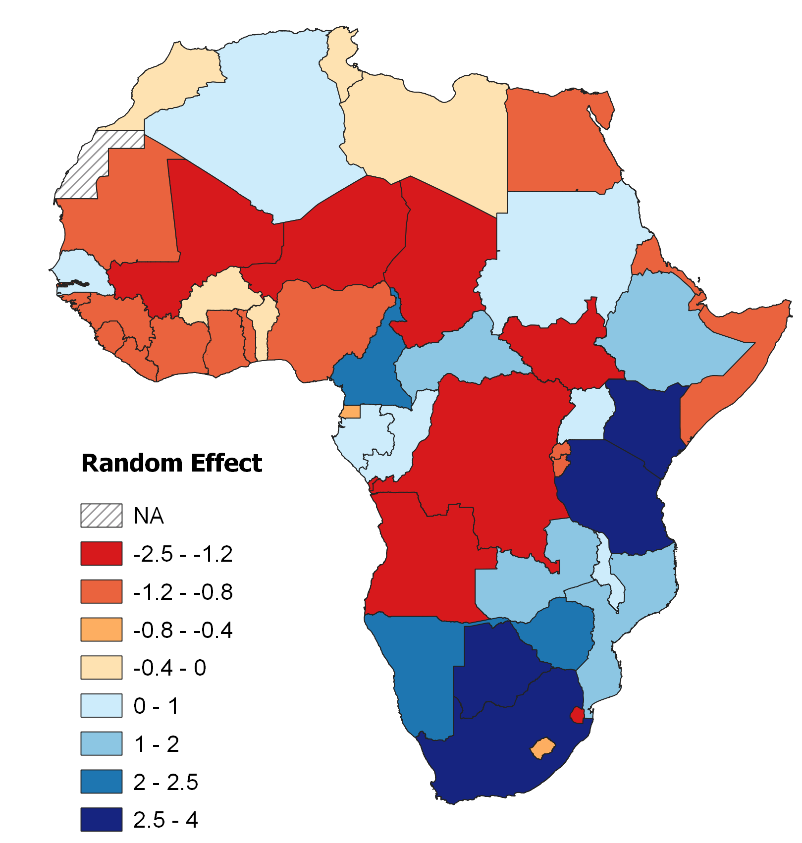


**Figure S1.** Random effects for individual countries from the Poisson GLMM investigating biases in large carnivore population assessments in Africa (species and countries only, not accounting for differences in large carnivore geographical range between countries or other variables; Model 1). A positive value (blue) indicates more population assessments (all species) than expected, and a negative value (red) indicates fewer. See Table S9 for country specific values.

Appendix S5 – Additional model fitting details

**All species Poisson GLMM**

Model dispersion (0.70) was appropriate, and the posterior predictive check indicated good model fit (Fig. S2).


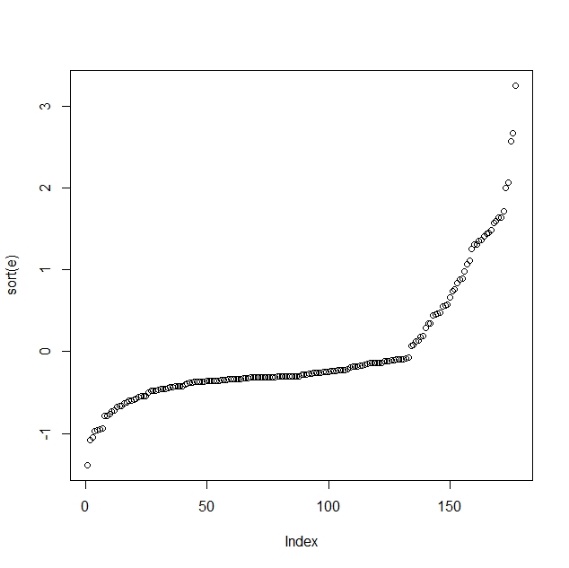

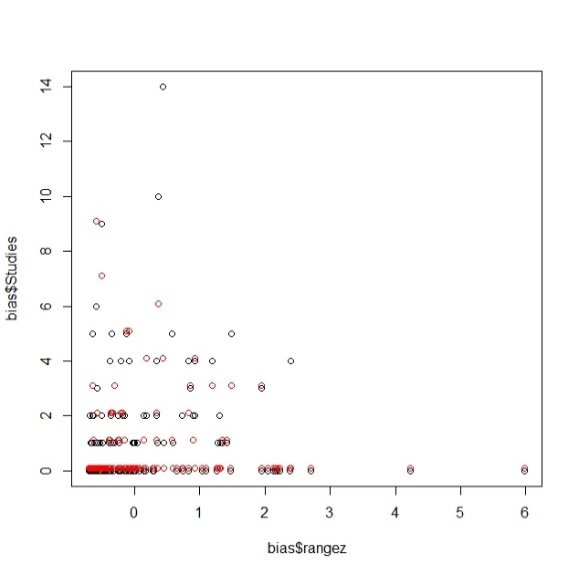


**Figure S2.** Model fit and posterior predictive check (PPC) for the most parametrised GLMM. The latter shows the actual data (black) and simulated data from the model (red). The check suggests a good fit.

**Lion Poisson GLM**

A Poisson GLM was first fitted to the data. This was overdispersed (dispersion parameter = 3.20 with no outliers removed, and 2.37 with one removed), and the posterior predictive check (PPC) showed that the model did not fit the observed data well. The data were not better fitted by a zero-inflated Poisson (ZIP) GLM. On the other hand, fitting a negative binomial (NB) GLM showed only little evidence of overdispersion (dispersion parameter = 1.52) and the PPC shows the model fitted the data well (Fig. S3); results from this are therefore presented in text.


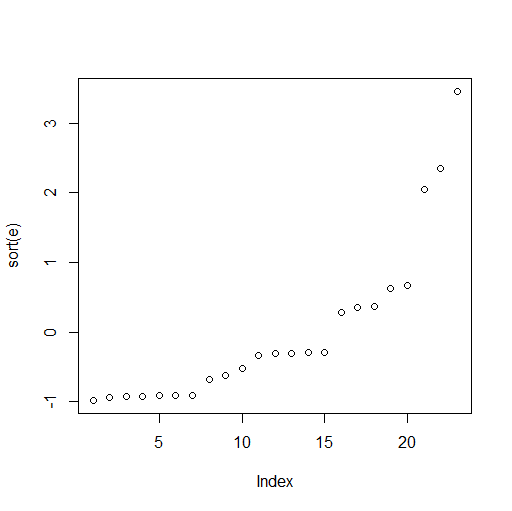

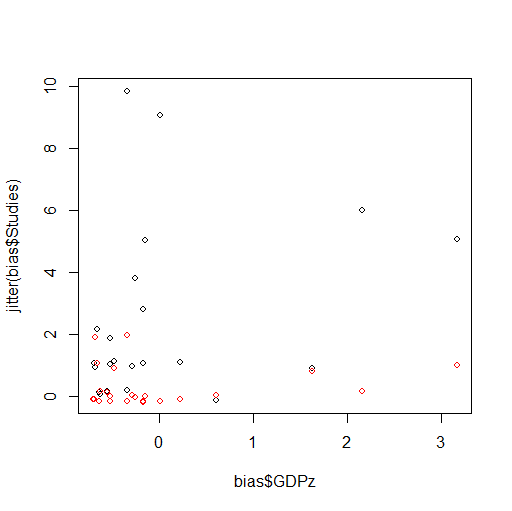


**Figure S3.** Model fit and posterior predictive check (PPC) for the NB GLM (Range) for lion, showing the actual data (black) and simulated data from the model (red). The check indicates slight overdispersion (dispersion parameter = 1.52), and the PPC suggests the model fitted the observed data well.

**Leopard Poisson GLM**

Leopard data were first fitted as a Poisson GLM (N=41). This was overdispersed (dispersion parameter = 4.46). Once the outlier (South Africa) was removed, the model fitted the data well with little evidence of overdispersion (dispersion parameter = 1.13; Fig. S4); as a result, outputs from this model are presented.


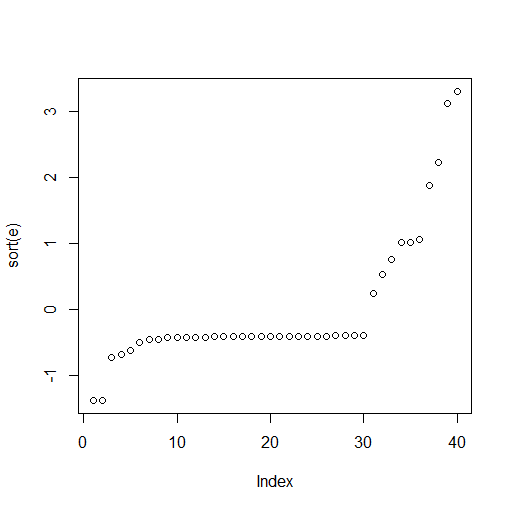

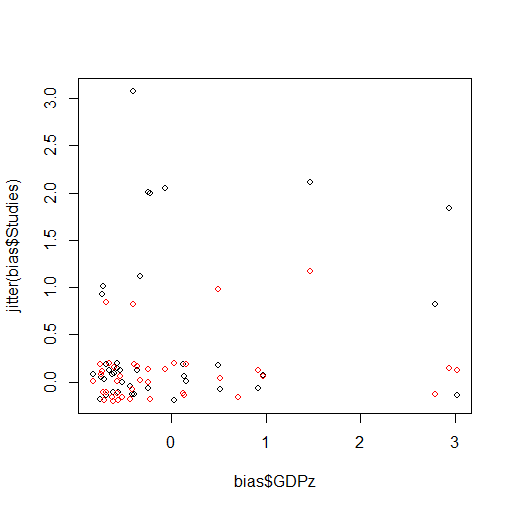


**Figure S4.** Model fit and posterior predictive check (PPC) for the Poisson GLM (range only) for leopard, with one outlier removed (South Africa), showing the actual data (black) and simulated data from the model (red). The check indicates almost no overdispersion (dispersion parameter = 1.13), and the PPC suggests the model fitted the observed data well.

**Spotted Hyaena Poisson GLM**

Spotted hyaena data were fitted as a Poisson GLM (N=40). There was some overdispersion (dispersion parameter = 2.33), but no outliers. The PPC revealed the model fitted the data relatively well (Fig. S5); as a result, outputs from this model are presented.


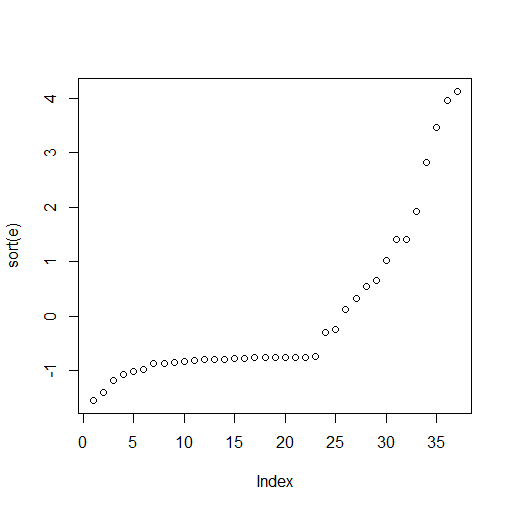

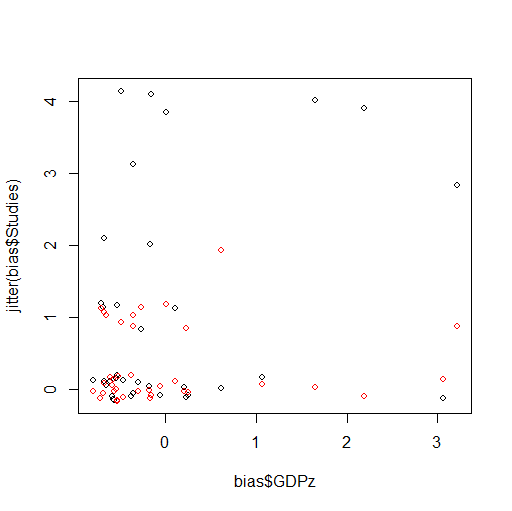


**Figure S5.** Model fit and posterior predictive check (PPC) for the Poisson GLM for spotted hyaena, showing the actual data (black) and simulated data from the model (red). The check indicates some overdispersion (dispersion parameter = 2.33), with the PPC suggesting the model fitted the observed data relatively well.

**Cheetah Poisson GLM**

Cheetah data were fitted as a Poisson GLM (N=20). There was slight overdispersion (dispersion parameter = 1.72), but no outliers. The PPC revealed the model fitted the data relatively well (Fig. S6); as a result, outputs from this model are presented.


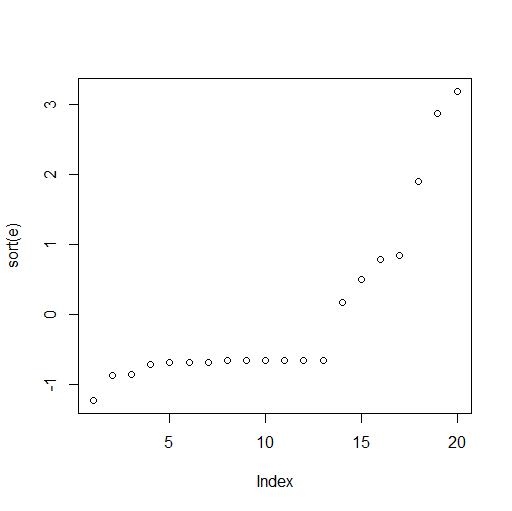

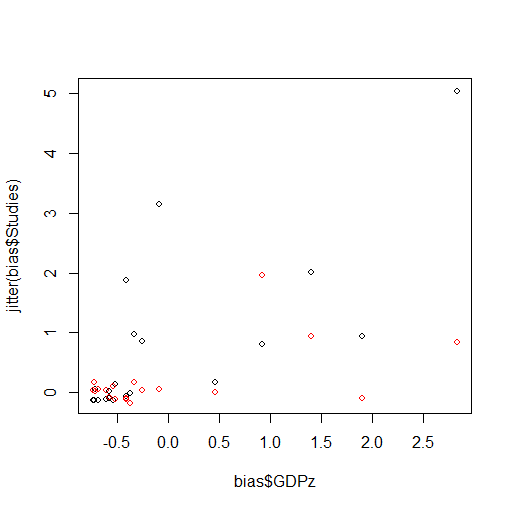


**Figure S6.** Model fit and Posterior predictive check (PPC) for the Poisson GLM for cheetah, showing the actual data (black) and simulated data from the model (red). The check indicates slight overdispersion (dispersion parameter = 1.72), with the PPC suggesting the model fitted the observed data relatively well.
